# Contribution of vitamin D_3_ and thiols status to the outcome of COVID-19 disease in Italian pediatric and adult patients

**DOI:** 10.1038/s41598-023-29519-7

**Published:** 2023-02-13

**Authors:** Annamaria D’Alessandro, Domenico Ciavardelli, Anna Pastore, Santina Lupisella, Rosa Carmela Cristofaro, Giovina Di Felice, Roberta Salierno, Marco Infante, Alberto De Stefano, Andrea Onetti Muda, Maria Morello, Ottavia Porzio

**Affiliations:** 1 Clinical Biochemistry Laboratory, IRCCS Bambino Gesù Children’s Hospital, 00165 Rome, Italy; 2 School of Medicine, University “Kore” of Enna, 94100 Enna, Italy; 3 Center for Advanced Studies and Technology (C.A.S.T.), G. D’Annunzio University of Chieti-Pescara, 66100 Chieti, Italy; 4 Research Unit of Diagnostical and Management Innovations, IRCCS Bambino Gesù Children’s Hospital, 00165 Rome, Italy; 5 Clinical Biochemistry Department, Tor Vergata University Hospital (PTV), Rome, Italy; 6 Department of Systems Medicine, Diabetes Research Institute Federation (DRIF), Tor Vergata University, Rome, Italy; 7 UniCamillus, Saint Camillus International University of Health Sciences, Rome, Italy; 8 Network of Immunity in Infection, Malignancy and Autoimmunity (NIIMA), Universal Scientific Education and Research Network (USERN), Rome, Italy; 9 Psychiatric Unit Department of Systems Medicine, Tor Vergata University, Rome, Italy; 10 Volunteers Association of Tor Vergata University Hospital (PTV), Rome, Italy; 11 Clinical Biochemistry and Molecular Biology, Department of Experimental Medicine, Faculty of Medicine, Tor Vergata University, Rome, Italy; 12 Department of Experimental Medicine, Tor Vergata University, Rome, Italy

**Keywords:** Diagnostic markers, Liquid chromatography, Biochemical assays

## Abstract

The coronavirus disease 2019 (COVID-19), caused by severe acute respiratory syndrome coronavirus 2 (SARSCoV-2), was declared a global pandemic by the World Health Organization (WHO) on March 2020, causing unprecedented disease with million deaths across the globe, mostly adults. Indeed, children accounted for only a few percent of cases. Italy was the first Western country struck by the COVID-19 epidemic. Increasing age, which is one of the principal risk factors for COVID-19 mortality, is associated with declined glutathione (GSH) levels. Over the last decade, several studies demonstrated that both vitamin D (VD) and GSH have immunomodulatory properties. To verify the association between VD, GSH and the outcome of COVID-19 disease, we conducted a multicenter retrospective study in 35 children and 128 adult patients with COVID-19. Our study demonstrated a hypovitaminosis D in COVID-19 patients, suggesting a possible role of low VD status in increasing the risk of COVID-19 infection and subsequent hospitalization. In addition, we find a thiol disturbance with a GSH depletion associated to the disease severity. In children, who fortunately survived, both VD and GSH levels at admission were higher than in adults, suggesting that lower VD and thiols levels upon admission may be a modifiable risk factor for adverse outcomes and mortality in hospitalized patients with COVID-19.

## Introduction

The coronavirus disease 2019 (COVID-19), caused by severe acute respiratory syndrome coronavirus 2 (SARSCoV-2), was first reported in December 2019 in Wuhan, a city in China. COVID-19 was then declared by the World Health Organization (WHO) a global pandemic (March 11, 2020). Italy was the first Western country struck by the COVID-19 pandemic. COVID-19 continues to cause unprecedented disease with medical, social, and economic challenges across the globe. As of 6th June 2022, more than 531 million cases have been confirmed worldwide, with over 6.3 million deaths (https://coronavirus.jhu.edu/map.html), mostly adults. Indeed, for much of the past 2 years, children accounted for only a few percent of cases, less than 5% in most areas, with hospitalization rates below 2% and mortality rate under 1%. Several risk factors, including age, hypertension, ischemic heart disease, diabetes, and chronic respiratory disease have been identified to be associated with a high mortality rate. These heterogeneous risk factors could be associated with a common pathway that may cause a high incidence of COVID-19 mortality.

Increasing age, which is one of the most noticeable risk factors for COVID-19 mortality, is associated with declined glutathione (GSH) levels^1,2^. This decline may be due to extensive GSH oxidation or a combination of extensive GSH oxidation and a decrease in the total pool of thiol^1^. The drop in the thiol pool was also reported in a larger scale study in middle-aged and older community-living healthy subjects in Europe^3^. Under either circumstance, this will lead to a drop in the level of available GSH.

During the last decade, different studies have shown that vitamin D (VD) possess anti-inflammatory and immunomodulatory property^4^. VD comprise several forms of the vitamin. Two forms important in humans are vitamin D_2_ (VD_2_) (ergocalciferol) and vitamin D_3_ (VD_3_) (cholecalciferol). VD_2_ is mainly of plant, fungal, and/or yeast origin, whereas VD_3_ is the form synthesized in the skin in the presence of B ultraviolet rays (UVB), although it may also have this dietary origin. Both VD_2_ and VD_3_ are biologically inactive forms of VD, and, before becoming active into body, they must be converted to the active forms in liver and kidney. VD plays a pivotal role in the regulation of both innate and adaptive immune responses by interacting with its receptor (VDR). VDR is indeed expressed by several cells, including immune cells. VD is able to promote the synthesis of antiviral effectors and modulates the expression of pro-and anti-inflammatory cytokines^5,6^. There is a strong association between risk factors for VD deficiency and severe COVID-19 (such as Black or Asian ethnic origin, older age, and obesity)^7^. For this reason, over the last period, a number of studies have suggested that VD deficiency is an independent risk factor for COVID-19 infection and unfavorable outcomes^8–10^. Similarly, interest is growing about the potential role of VD as an immunomodulatory adjuvant able to prevent COVID-19 infection or work against the development of the cytokine storm, improving the outcome of the COVID-19 disease^9,11^.

In a foresight study, that precedes the COVID-19 outbreak, Jain and colleagues demonstrated that VD deficiency is associated to lower GSH levels and that the boost of GSH status up-regulates the genes of VD metabolism and VDR. Indeed, both VD metabolism and VDR genes are required to raise the bioavailability and the blood levels of VD, favoring the reduction of inflammation^12^.

In order to verify whether VD and thiols status are associated to the outcome of COVID-19 disease, we conducted a multicenter retrospective study among both children and adult patients with COVID-19 admitted to our Institutions during the first wave of the Italian COVID-19 outbreak. The study endpoints were: (1) to determine VD_2_, VD_3_ and total VD in COVID-19 children and adults; (2) to compare the levels of VD and thiols concentrations between adult Survivor (S), adult Non-Survivor (NS) and our internal reference values, and between children (C) and our internal reference values; (3) to assess the potential protective role of VD and thiols against the progression and severity of COVID-19 infection.

## Results

Table 1 shows the demographical and clinical data of COVID-19 patients at admission. Although the mean age of NS patients shows a trend toward higher age, S and NS patients are matched for age. In contrast, NS and S groups differ for gender % with a prevalence of males in the NS compared with S patients. Several deceased patients had some co-morbidity (hypertension, obesity (defined as body mass index, BMI, > 30), and/or diabetes). However, the comorbidity prevalence does not significantly differ among the NS and S groups. As expected, the comorbidity prevalence was higher in NS and S patients compared with C patients. All the inflammation marker levels are higher in deceased patients when compared to survived adults. Of note, neutrophil-to-lymphocyte ratio (NLR), an emerging marker of systemic inflammation^13^, is significantly higher in NS compared to S. C-reactive protein (CRP) levels were also dramatically increased in our deceased patients when compared to S. All other inflammation markers, such as D-dimer, fibrinogen, procalcitonin (PTC), lactate dehydrogenase (LDH), interleukin 6 (IL-6), and tumor necrosis factor alpha (TNF-α) were significantly increased in NS compared to S patients (Table 1). Regarding our pediatric cohort, fortunately all patients survived. All the inflammatory markers were only slightly increased in C when compared to reference values, and significantly lower when compared with NS and S patients.

**Table 1:** General characteristics of the study cohorts.

Variable	Adult		Children (C)	*p*^a^
	Non-survivors (NS)	Survivors (S)		
N	51	87	35	
Age, y, mean (standard deviation)	69 (15)	65 (13)	7 (5)	NS vs S = 0.30 S vs C < 0.001
Gender, male (%)	39 (76.47)	41 (47.13)	19 (54.28)	NS vs S = 0.025 S vs C = 0.35
Comorbidities (%)	37 (72.54)	42 (48.27)	6 (17.14)	NS vs S = 0.17 S vs C = 0.013
NLR ratio, median (interquartile range)	12 (17)	4 (6)	1.0 (0.8)	NS vs S < 0.001 S vs C < 0.001
CRP, mg/dL, median (interquartile range)	125 (150)	44 (115)	0.1 (0.4)	NS vs S < 0.001 S vs C < 0.001
D-dimer, μg/mL FEU, median (interquartile range)	1927 (10,433)	844 (1043)	0.5 (0.7)	NS vs S < 0.001 S vs C < 0.001
Fibrinogen, mg/dL, mean (standard deviation)	683 (270)	543 (284)	323 (80)	NS vs S = 0.002 S vs C < 0.001
IL-6, pg/mL, median (interquartile range)	73 (182)	28 (66)	27 (17)	NS vs S = 0.002 S vs C = 0.14
TNF-α, pg/mL, median (interquartile range)	21 (20)	15 (21)	16 (22)	NS vs S = 0.017 S vs C = 0.80
PCT, ng/mL, median (interquartile range)	1 (2)	0.1 (0.7)	0.07 (0.06)	NS vs S < 0.001 S vs C = 0.008
Ferritin, ng/mL, median (interquartile range)	1259 (2227)	549 (1013)	61 (82)	NS vs S < 0.001 S vs C < 0.001
LDH, mU/mL, median (interquartile range)	444 (282)	284 (170)	260 (113)	NS vs S < 0.001 S vs C = 0.15

Data are expressed as mean ± standard deviation (SD) or median ± interquartile range (IQR).

^a^Calculated using one-way ANOVA followed by Bonferroni post-hoc test or Kruskal–Wallis test followed multiple comparison of mean ranks for continous variables and χ^2^ test for categorical variables.

Table 2 shows the levels of VD in our patients. To our knowledge, this is the first study reporting the analysis of the two principal forms of VD, namely VD_2_ and VD_3_, in the plasma from patients affected by COVID-19. As VD_3_ is the form produced in the human skin, it is thus not surprising that only VD_3_ is significantly lower in adults compared to children. This is more evident in NS than in S patients. The desirable levels of total VD are ranging from 30 to 50 ng/mL^14^. Our total VD values are indeed severely low in NS, low in S, and only insufficient in children. Therefore, although reduced when compared with the lower reference value for the total VD, VD levels directly correlate with the severity of the disease in our study populations.

**Table 2:** Comparative VD levels between adult non-survivors, adult survivors and children.

Analyte	Adult non-survivors (NS)	Adult survivors (S)	Children (C)	*p*
VD_2_, nmol/L	1.6 (0.4)	1.5 (0.5)	2 (1)	NS vs S = 0.070 S vs C < 0.001
VD_3_, nmol/L	16 (22)	27 (25)	50 (32)	NS vs S = 0.040 S vs C < 0.001
Total VD, nmol/L	19 (22)	28 (26)	53 (32)	NS vs S = 0.021 S vs C < 0.001
Total VD, ng/mL	7.5 (9)	11.5 (10)	18.4 (11)	NS vs S = 0.028 S vs C < 0.001

Data are expressed as median (interquartile range, IQR).

**Figure 1:**
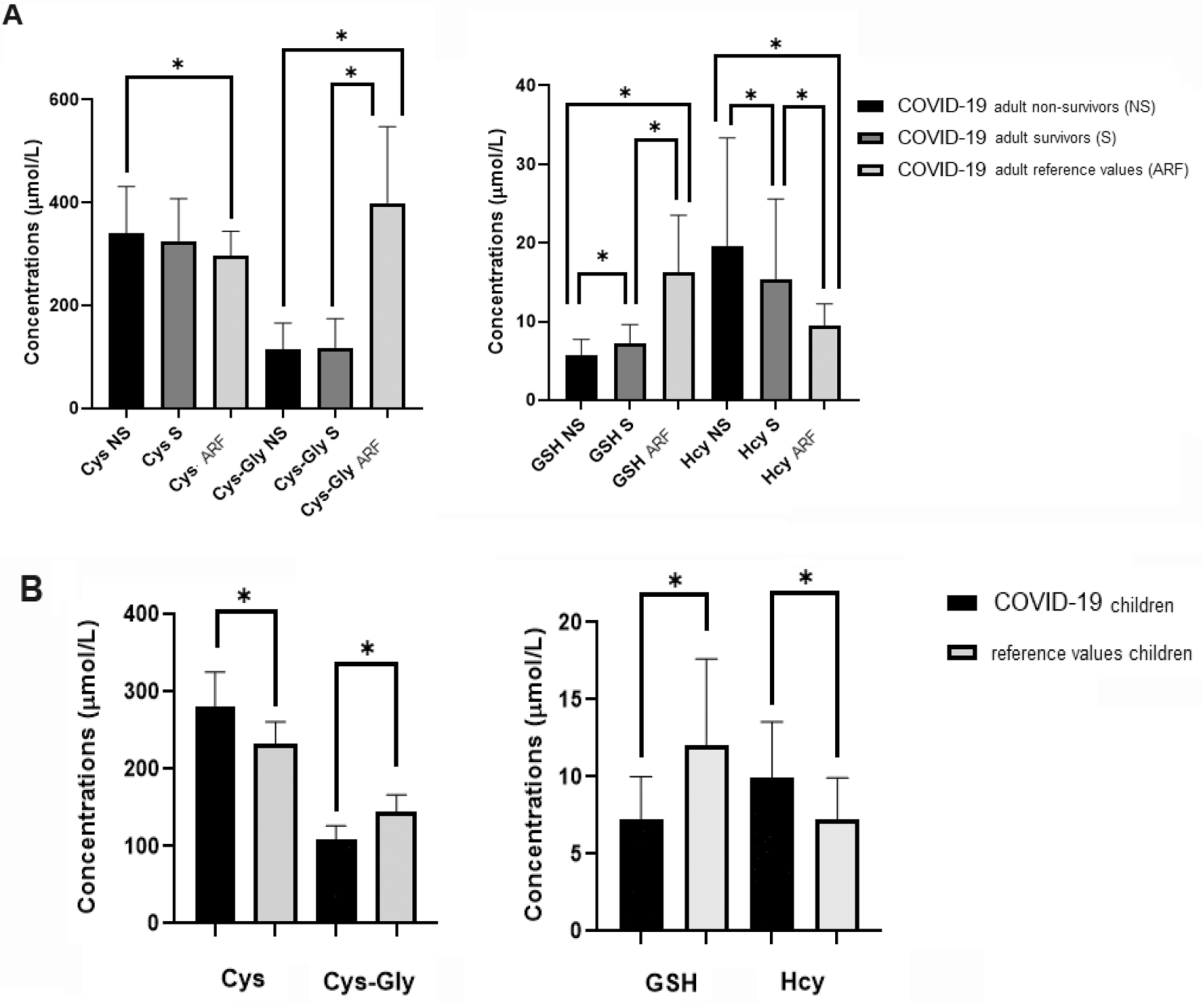
Low molecular weight amino thiols in adults and children. Low molecular aminothiols levels (LMWTs: cysteine (Cys), cysteinyl-glycine (Cys-Gly), glutathione (GSH), and homocysteine (Hcy)) in non-survivors (NS) and survivors (S) adults, compared to adult internal reference values (ARF) concentrations (panel **A**; *p < 0.050). LMWTs levels in children cohort compared to LMWTs children internal reference values (panel **B**; *p < 0.050).

Table 3 shows the thiols status of the patients at admission. It has been hypothesized that increasing age, which is one of the most noticeable risk factors for COVID-19 mortality, is associated with a decrease in the thiols pool^1^. Here we report an imbalance in almost all thiols in adult patients respect to our internal reference values and some values did correlate with the severity of the disease. In Fig. 1A are reported the thiol values in the adult cohort compared to our internal reference values. Cysteine (Cys) mean values are comparable between NS and S (*p* = 0.17), but are significantly higher compared to the internal reference values (*p* < 0.05). Cys-Gly mean values are also similar in NS and S patients (*p* = 0.19), but extremely lower than our internal reference value (*p* < 0.05). GSH levels are dramatically lower in both NS and S patients respect to our internal reference values (*p* < 0.001). Total homocysteine (Hcy) levels are increased in both NS and S patients compared to our internal reference values (*p* < 0.05), and those levels did correlate with the COVID-19 outcome. Thiols status in children is shown in Fig. 1B. In COVID-19 patients there is an increase of Cys levels (p < 0.001) and a decrease of Cys-Gly values (p < 0.001) compared to our internal reference values. Furthermore, a decrease of GSH (*p* < 0.001) and an increase of Hcy (*p* < 0.001) were observed in C, confirming thus that the GSH decrease and the Hcy increase are associated with the disease regardless from patient’s age.

**Table 3:** Comparative thiols levels between adult non-survivors (NS), adult survivors (S), and children (C).

Analyte	Adult non-survivors (NS)	Adult survivors (S)	Children (C)	Adults internal Reference Values (ARF)	Children internal Reference Values (CRF)	*p*
Cys	339 (84)	318 (77)	280 (45)	296 (48)	233 (31)	NS vs S = 0.17 NS vs ARF < 0.001 S vs ARF = 0.032 C vs CRF < 0.001
Cys-Gly	119 (58)	110 (28)	109 (17)	400 (148)	145 (21)	NS vs S = 0.69 NS vs ARF < 0.001 S vs ARF < 0.001 C vs CRF < 0.001
GSH	6 (2)	7 (2)	7 (3)	16 (7)	12 (6)	NS vs S < 0.001 NS vs ARF < 0.001 S vs ARF < 0.001 C vs CRF < 0.001
Hcy	15 (9)	12 (11)	10 (4)	9 (3)	8 (3)	NS vs = 0.034 NS vs ARF < 0.001 S vs ARF < 0.001 C vs CRF < 0.001

Values are expressed as μmol/L; mean (standard deviation), or median (inter-quartile range, IQR) are reported.

**Figure 2:**
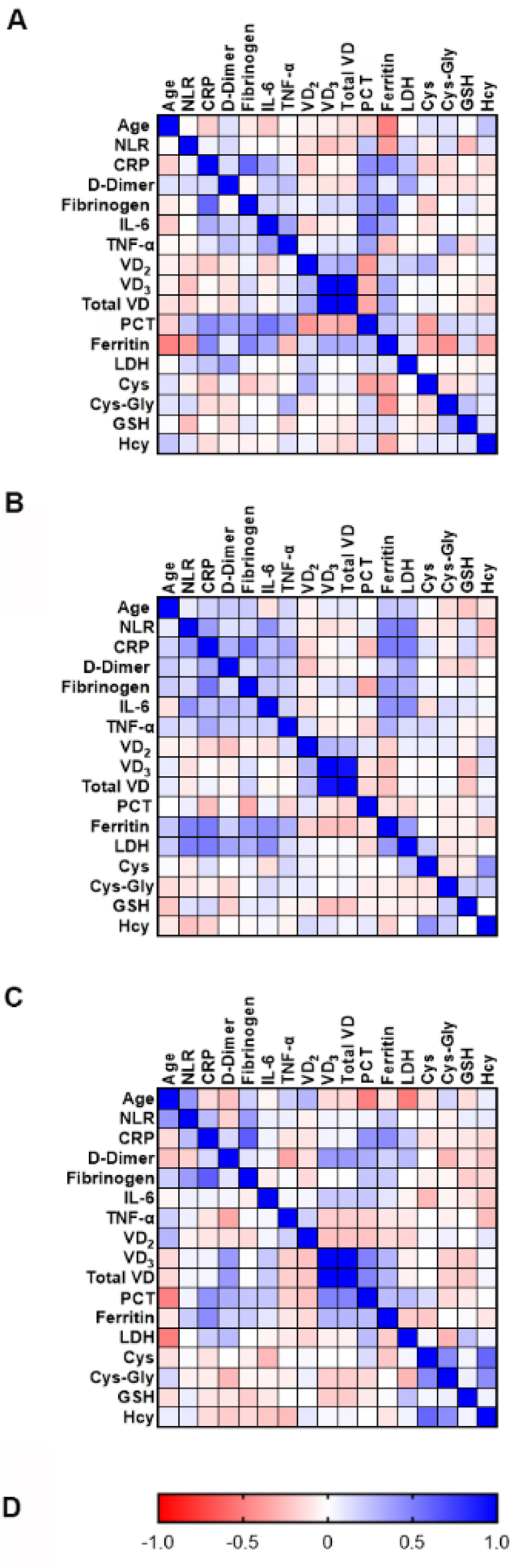
Spearman's correlation analysis of the measured clinical variables. The figure shows the heatmaps of the correlation between all the parameter studied in the adult non-survivors (NS, panel **A**), in the adult survivors (S, panel **B**), and in children (C, panel **C**). Spearman’s correlation coefficients vary according to the color scale shown in the panel (**D**).

The correlation heatmaps for the clinical features studied in the NS, S, and C patients are shown in Fig. 2A–C, respectively. The Spearman’s correlation coefficients (ρ) and the calculated p-values are shown in the Supplementary Tables 1–3. Among the significant correlations, we found that the level of VD3 positively correlate with total VD in all the study groups (Supplementary Tables 1–3). Furthermore, serum levels of PCT negatively correlate with serum levels of VD2, VD3, and total VD in the NS group. In contrast, serum levels of PCT positively correlate with serum levels VD3 and total VD in the C group. These correlations were not significant in the S group.

**Figure 3:**
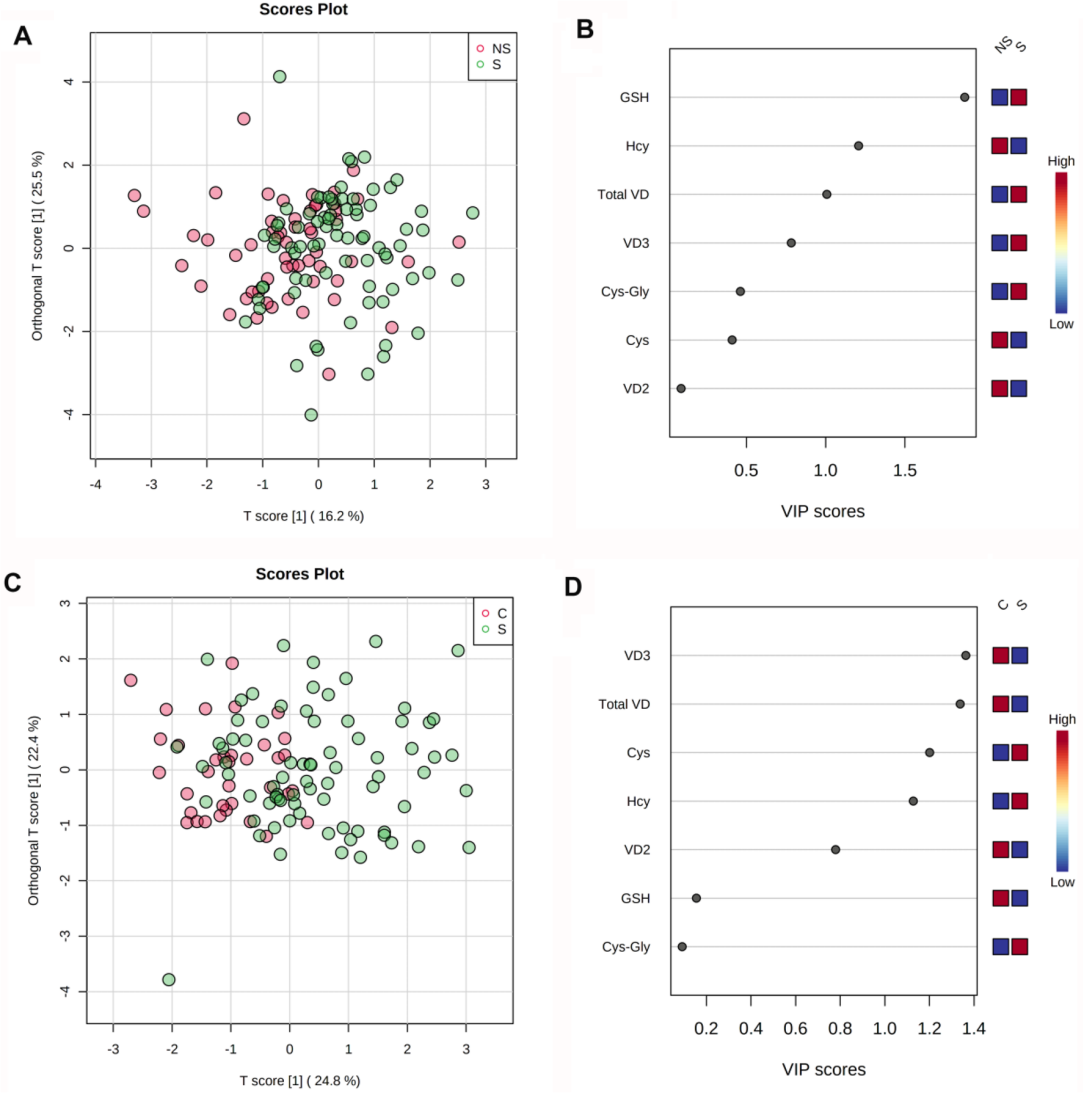
Orthogonal Partial Least Squares (OPLS-DA) analysis of serum vitamin D and thiols in COVID-19 patients. The figure shows the OPLS-DA score plot and Variable Importance in Projection (VIP) score-plot of the OPLS-DA model calculated to discriminate between non-survived (NS) and survived (S) adult patients (**A**, **B**), and S and children (C) (**C**, **D**) patients**.**

In order to further investigate the specific potential role of VD and thiols status in the progression of disease we performed unsupervised and supervised multivariate analysis of VD2, VD3, total VD, and thiols levels. Unsupervised hierarchical cluster analysis shows that VD2, VD3, and total VD levels account for the separation between the C group and the NS and S groups (Supplementary Fig. 1). In contrast, the calculated model was not able to discriminate between NS and S groups (Supplementary Fig. 1). The supervised OPLS-DA support the idea that VD status is the main factor able to discriminate the C group from adults (Fig. 3). In fact, the VIP scores for VD3 and total VD were higher than 1 for the comparisons between S and C groups (Fig. 3C). On the other hand, the subtle discrimination between the NS and S groups seems to be related to the differences in GSH and Hcy (Fig. 3A). In fact, the levels of GSH are significantly lower in the NS patients compared with the S patients while Hcy levels were higher in the NS patients compared with the S patients. The VIP scores for these variables were both greater than 1 (Fig. 3B) indicating that both GSH and Hcy are relevant variable discriminating NS from S patients.

## Discussion

In our cohorts, NS patients showed statistically significant higher levels of inflammation, coagulation, and sepsis markers (such as NLR, CRP, ferritin, IL-6, TNF-α, D-dimer, fibrinogen, PCT, and LDH) compared to S patients. These findings agree to that reported recently in asymptomatic or mild to moderate COVID-19 patients as well as in patients with severe COVID-19^14^. In particular, NLR, CRP, ferritin, D-dimer, fibrinogen, PCT and LDH levels are sharply altered when comparing our adults and C cohorts, endorsing the milder form of the disease found in children. Regarding IL-6 and TNF-α, S and C patients are instead surprisingly similar. IL-6 is a pleiotropic inflammatory cytokine that is produced transiently due to tissue damage and infections. IL-6 has a broad effect on cells of the immune system and could have context-dependent pro- and anti-inflammatory properties^15^. Is thus possible that IL-6 levels are higher when the inflammation is massive, as in our NS patients, and lower when the inflammation is under control, as in both S and C patients, where it could exert its anti-inflammatory role. TNF-α is an extremely multifaceted intermediary, with structurally distinct domains that could function in either a damaging or a defensive way^16^. When the concentrations of TNF-α are higher, TNF-α work as regulator of lung function in deleterious manner, by inducing hyper-inflammation. However, as reviewed by Lucas et al.^16^, in some pathological conditions, such as ventilator-induced lung injury, the two TNF receptors can intercede contrasting effects after binding TNF-α, with TNF Receptor 1 being damaging and TNF Receptor 2 slightly protective. Furthermore, the TNF lectin-like domain, which is different from the receptor 1 and 2 binding sites, is mostly protecting, reducing inflammation when activated. It is thus possible that, in S and C patients, TNFα levels were quite similar because of the dual role of this important cytokine^16^.

NLR is a recognized indicator of systemic inflammation^13^ and a suitable marker for hospital mortality in patients with acute worsening of chronic obstructive pulmonary disease (COPD)^17^. Recently, Gan and collaborators^18^ demonstrate that NLR could be an efficient indicator of mortality in hospitalized patients with COVID-19. In the same way, Hu and colleagues^19^ showed that admission levels of PCT could also help in outlining the disease severity and death in hospitalized COVID-19 patients. It was recently demonstrated that PCT-related inflammation alters mitochondrial bioenergetics in early critical illness mainly associated to nutrient deprivation and oxidative stress^20^. A negative correlation between PCT levels and VD status was also demonstrated in patients with Ventilator Associated Pneumonia^21^. In this context, VD administration correlated with the reduced mortality rate of patients with VAP. Accordingly, we found a negative correlation between PCT and VD levels in our NS patients. This negative correlation disappears in S patients and became positive in C patients, thus confirming the association between VD, PCT and mortality in COVID-19 disease.

In addition, our study endorses the role of low VD levels as a potential risk factor for COVID-19 infection and hospitalization. Using LC–MS/MS, currently considered the gold standard for the measurement of VD metabolites, we demonstrated for the first time that in COVID-19 patients VD_3_, and not VD_2_, is the mainly form of VD correlated to the improvement of inflammatory status. We found low VD levels in our adult cohorts at admission. Our findings agree to those observed in previous studies by our group and by other authors. In a small adult population, we have recently demonstrated that low total VD levels were associated with higher risk of mortality in COVID-19 patients, independent of age, sex, markers of inflammation, coagulation, sepsis, and co-morbidities^8^. Accordingly, it was recently demonstrated in a large population-based study that there is an independent and significant association between low VD levels (< 30 ng/mL) and the increased probability of COVID-19 infection^22^. Moreover, in a smaller retrospective cohort study significantly lower VD levels were found in COVID-19 patients compared to controls^23^. In addition, in a retrospective observational analysis on a bulky group of COVID-19 patients, it was demonstrated that the COVID-19 positivity rate was higher in patients with low VD values (< 20 ng/mL) compared to patients with sufficient values^24^. Indeed, total VD levels in our adult cohort were significantly higher in S than in NS. Accordingly, a retrospective observational study on 42 hospitalized patients with COVID-19-related acute respiratory failure shows that severe VD deficiency (defined as VD < 10 ng/mL) is associated to significantly higher mortality risk^25^. In another retrospective study on 185 COVID-19 patients, VD deficiency (defined as VD < 12 ng/mL) was associated with higher risk of invasive mechanical ventilation and death^26^. Taken together, those results may suggest the existence of a threshold of VD level, which might predict poor clinical outcomes in COVID-19 patients. According with these studies and in agreement with our data, this level is ranging between 8 and 12 ng/mL^8^. Indeed, we demonstrated that: (1) in our adult COVID-19 patients, low VD status contributes to the worsening systemic inflammation among NS. Conversely, (2) a sufficient VD levels measured in adult patients increases survival and probably improves recovery from disease, and (3) higher VD concentration, measured in the children cohort, contributes to mitigate the symptoms of SARSCov-2 infection. This could be reasonable considering the anti-inflammatory and immunomodulatory properties of VD^5,6^. Accordingly, in a retrospective case–control study, Hernandez et al.^27^ found that VD levels were significantly lower in 216 hospitalized COVID-19 patients compared to 197 sex-matched controls (14 ± 7 vs. 21 ± 7 ng/mL, respectively). COVID-19 patients also showed a higher prevalence of VD deficiency (defined as VD < 20 ng/mL), compared to healthy subjects (82% vs. 47%). Moreover, low VD levels shows a significant correlation with high ferritin and D-dimer levels. Accordingly, Ye and collaborators^28^ found significantly lower VD levels in COVID-19 hospitalized patients compared to controls. In addition, a significantly higher number of subjects with VD deficiency (defined as VD < 20 ng/mL), was found in COVID-19 patients compared to controls^28^.

It has been previously demonstrated that a precise disulfide-thiol balance is critical for viral entry and fusion to the host cell, and that oxidative stress engendered from free radicals can negatively affect this balance^29^. Low molecular weight amino thiols (LMWTs) such as Cys, Cys-Gly, GSH, and Hcy play a fundamental function in the biochemical processes implicated in the body’s response to SARSCov-2 infection^1,2^. Therefore, they could be potential biomarkers of the harshness of the COVID-19 disease. GSH is the main intracellular non-enzymatic antioxidant and protein glutathionylation is one of the important mechanisms of posttranslational regulation of proteins function^30^. Low GSH levels are linked with a tendency to infections of the respiratory tract^31^. Furthermore, Jain and colleagues demonstrated that VD deficiency is associated to lower GSH levels^12^. Therefore, it was hypothesized that GSH deficiency, together to VD deficiency, could play a central role in the COVID-19 pathophysiology. Indeed, GSH is crucial for the performance of the immune system, especially T lymphocytes and macrophages, and is responsible for proper T lymphocyte function, proliferation, and prevention of apoptosis. Moreover, GSH has an inhibitory effect on many viral strains. Accordingly, it has been recently reported that low GSH levels could be regarded as a marker for the development of severe COVID-19 disease^32–35^. Our results agree with those reported, as GSH levels are significantly decreased in both adult and pediatric patients respect to our internal reference values. In addition, our data suggest that the GSH reduction correlate with the severity of the COVID-19 disease. This agrees with the conclusions of Kryukov and collaborators^32^, which find an association between low molecular weight thiols and severity of COVID-19 disease. Regarding the others LMWTs, we found comparable levels of Cys in adults and higher Cys levels in C patients, respectively, compared to the reference values. Furthermore, GSH levels did not significantly correlate with Cys levels in all study groups. Therefore, the decrease of GSH levels in COVID-19 patients is not paralleled by a decrease of serum Cys. This finding could be quite surprising as Cys is a precursor of GSH through the pathway involving the enzymes γ-glutamylcysteine synthetase (γ-GCS) and glutathione synthetase (GS)^36^. However, the lack of correlation between serum GSH and Cys can be explained by inhibition of the γ-GCS activity induced by corticosteroids used to counteractearly inflammation in COVID-19 patients. Indeed, it was previously demonstrated that dexamethasone decreased GSH levels in alveolar epithelial cells by down regulating the transcription of the γ-GCS-Heavy Subunit gene^37^. Hcy is obtained from methionine via the two intermediates S-adenosylmethionine and S-adenosylhomocysteine, and have a fundamental role in the regulation of cytokine and inflammatory protein gene expression as well as in the production of viral particles. In this study, we found higher Hcy levels in NS and S patients compared to our internal reference values, whereas physiological levels of Hcy have been found in C patients. In addition, NS shows higher Hcy levels compared to S. Accordingly, in a large study of 273 patients with COVID-19, the lungs disease development, as revealed by computerized tomography (CT), was associated with the level of Hcy, hypothesizing thus a role of Hcy in the progression to severe COVID-19^38^. Most recently, in a multicenter, retrospective analysis on Covid-19 hospitalized patients it was demonstrated that Hcy is a predictive marker for COVID-19 disease outcome, as plasma Hcy levels correlate significantly with COVID-19 severity, with an optimal cut-off value of 16 μmol/L. They also pointed out that the supplementation with vitamin B9 and other vitamins of the same group (for example B12) has been demonstrated to normalize blood Hcy levels, suggesting thus that proper integration of vitamin B and Folic acid could have protective clinical effects in patients with infectious disease^39^.

It has been recently shown that paracetamol (PAC), extensively adopted in the therapy of COVID-19 disease, could be a link between GSH consumption and the severe COVID-19 illness^40^. In fact, as discussed above, therapeutic doses of PAC can lower GSH levels, thus impairing the endogenous antioxidant defenses. Furthermore, an illuminating commentary suggests that is important than prescription of PAC should be endorsed with caution in sensitive populations with scarce GSH levels, such as the elderly^41^. It was recently demonstrated that a parallel intake of *N*-acetyl-cysteine (NAC) could be useful in order to correct the GSH deficiency in this population^42^. In addition, it has clearly evidenced that GSH levels directly influence VD status^12^. Moreover, before the COVID-19 outbreak, a serious VD deficiency in the general population was reported^17^. We could therefore speculate that the current situation resembles a pandemic in the pandemic and that the generous use of paracetamol could exacerbate this scenario leading to a pandemic GSH deficiency^40^.

In conclusion, our study found a high prevalence of hypovitaminosis D in COVID-19 patients, suggesting a possible role of low VD status in increasing the risk of COVID-19 infection and subsequent hospitalization. In particular, by using LC–MS/MS technique it was possible to separate the VD_2_ and VD_3_ forms and calculate the total VD levels, allowing thus for the first time the identification of VD_3_ as the mainly VD form directly implicated in the protective effects on COVID progression and outcomes. In addition, in adult patients we find a thiol equilibrium disturbance, with a GSH depletion associated directly to the disease severity. We also found that deceased adult exhibited significantly lower VD levels at admission compared to both S and C patients, as well as higher levels of markers of inflammation, coagulation, and sepsis. In children, that are fortunately all survived, VD levels at admission were higher than in adults, but still inadequate. We therefore suggest that a lower VD status upon admission may be a modifiable risk factor and early predictive marker for adverse outcomes and mortality in hospitalized patients with COVID-19.

Our findings support the needing of future studies in order to gain further insights on the role of redox disturbances in COVID-19 infection, and trials to understand whether fighting both VD and GSH deficiency in the general population can prevent COVID-19 disease and counteract mortality.

## Materials and methods

### Study design

This multicenter retrospective cohort study including patients with confirmed COVID-19 infection was conducted during the first wave of the COVID-19 outbreak, between March and May 2020, when Italy was in strict lockdown.

The study population included 35 consecutive children from the Emergency Unit of “Bambino Gesù” Children’s Hospital and Research Institute of Rome, Italy, and 138 consecutive adults from the Emergency Unit of “Tor Vergata University Hospital” of Rome, Italy. Inclusion criteria for both adults and pediatric patients was the positivity to the SARS-CoV-2 nasopharyngeal swab qPCR test, regardless the severity of the disease. Exclusion criteria was the negativity of the qPCR test even in the presence of symptoms.

The estimated sample size was calculated according to values reported in a previous study^43^. In order to achieve a power of 80% with an alpha error rate of 0.05 the minimum sample size was approximately 52 patients.

This study complies with the Declaration of Helsinki and was performed according to the University of Rome Tor Vergata Ethics Committee approval (Registration Number: 141/20, July 23, 2020). All adult patients give written informed consent to anonymous data collection and analysis for research purposes. For children, informed consent was obtained from the parents of each child after illustrating the purpose and the nature of the study.

Demographic, medical history, blood test results, and outcomes were collected. Adult patients were divided into two outcomes groups, defined as S for the survived patients and NS for the dead patients.

### Measurements

All hematological and biochemical parameters were measured at admission. Thiols, VD forms, and cytokine levels were assessed in duplicate in the same center on sample collected from all patients. The other hematological parameters were measured using the same method in different clinical laboratory.

#### Thiols and vitamins D_2_ and D_3_ determinations

Plasma thiols were analyzed as previously reported^44^. For plasma VD_2_ and VD_3_ determinations, we use the MassTrak Vitamin D Solution from Waters, together with the ACQUITY UPLC I-Xevo TQ-S micro IVD (Waters Chromatography Europe BV, Etten-Leur, The Netherlands). Thiol levels of patient’s groups were compared with appropriate Adults and Children internal Reference Values (ARF and CRF, respectively) assessed by analyzing plasma collected from 103 gender and age matched healthy children and 77 gender and age matched healthy adults.

#### Cytokine determinations

Chemiluminescence method was used to measure IL-6 serum levels (reference range: 0–50 pg/mL; IMMULITE 2000 instrument, Siemens, Milan, Italy). Enzyme-linked immunosorbent assay (ELISA) technique was used to analyze TNF-α serum levels (reference range: 0–12.4 pg/mL; DRG, International Instruments GmbH, Marburg, Germany).

#### Other laboratory assays

An infectious disease specialist made the initial diagnosis of COVID-19 based on clinical symptoms (cough, fever, and dyspnea), and confirmed by positive SARS-CoV-2 nasopharyngeal swab qPCR test. Hematological and biochemical parameters were measured on blood, serum, or plasma samples collected at admission. Although the two Hospitals uses different analytical instrumentation, the analytical methods are identical. An automated hematological analyzer was used to measure the white blood cell (WBC) count. We also calculated the NLR, a marker of systemic inflammation^13^. An immunoturbidimetric method were used to measure the serum levels of CRP. Serum levels of ferritin and PCT were measured using chemiluminescence methods. Serum LDH levels were assayed by an UV assay. Plasma fibrinogen was determined by using clotting methods, whereas D-dimer levels were measured by immunoturbidimetric methods.

#### Statistical analysis

The GraphPad Prism 9.0 software were used to calculate descriptive statistics data, including mean and standard deviation (SD), median and percentiles (GraphPad Software, San Diego, CA, USA). In order to verify if the data were normally distributed both the visual inspection of the graph distribution and the Kolmogorov–Smirnov test of normality were used. Homoscedasticity of data was assessed using the Levene test. In the case of a normal distribution and homogeneity of the variance, significance of differences was assessed one way analysis of the variance (ANOVA) followed by the Bonferroni post-hoc test. When the assumptions of normality and homoscedasticity cannot be met, the Kruskal–Wallis test followed by the multiple comparison of the mean ranks was used. In both cases the Statistica 6.0 software (Statsoft, Tulsa, OK) was used. Significance of the differences between categorical variables such as gender and comorbidity prevalence were assessed using the χ^2^ test. The correlation between the clinical features collected for each study groups was studied using the Spearman’s rank correlation analysis. *p* values < 0.050 were considered statistically significant. Unsupervised hierarchical cluster analysis and supervised orthogonal partial least squares discriminant analysis (OPLS-DA) were performed using the Metaboanalyst 5.0 web server^45^ (accessed on August 2022) Variable Importance in Projection (VIP) scores greater than 1 was considered to select relevant variables in the models^46^.

### Institutional review board statement

The study was conducted in compliance with the Declaration of Helsinki, and was reviewed and approved by the Ethics Committee of University of Rome Tor Vergata (Registration Number: 141/20, July 23, 2020).

### Informed consent statement

All adult patients or the parents of each child provided written informed consent after illustrating the purpose and the nature of the study to anonymous data collection and analysis for research purposes.

## Supplementary Information

Supplementary Information.

## Data Availability

The authors confirm that the data supporting the findings of this study are available within the article. Raw data that support the findings of this study are available from the corresponding author, upon reasonable request.
