# Author Correction: Contribution of vitamin D3 and thiols status to the outcome of COVID-19 disease in Italian pediatric and adult patients

**DOI:** 10.1038/s41598-023-31323-2

**Published:** 2023-03-16

**Authors:** Annamaria D’Alessandro, Domenico Ciavardelli, Anna Pastore, Santina Lupisella, Rosa Carmela Cristofaro, Giovina Di Felice, Roberta Salierno, Marco Infante, Alberto De Stefano, Andrea Onetti Muda, Maria Morello, Ottavia Porzio

**Affiliations:** 1 Clinical Biochemistry Laboratory, IRCCS Bambino Gesù Children’s Hospital, 00165 Rome, Italy; 2 School of Medicine, University “Kore” of Enna, 94100 Enna, Italy; 3 Center for Advanced Studies and Technology (C.A.S.T.), G. D’Annunzio University of Chieti-Pescara, 66100 Chieti, Italy; 4 Research Unit of Diagnostical and Management Innovations, IRCCS Bambino Gesù Children’s Hospital, 00165 Rome, Italy; 5 Clinical Biochemistry Department, Tor Vergata University Hospital (PTV), Rome, Italy; 6 Department of Systems Medicine, Diabetes Research Institute Federation (DRIF), Tor Vergata University, Rome, Italy; 7 UniCamillus, Saint Camillus International University of Health Sciences, Rome, Italy; 8 Network of Immunity in Infection, Malignancy and Autoimmunity (NIIMA), Universal Scientific Education and Research Network (USERN), Rome, Italy; 9 Psychiatric Unit Department of Systems Medicine, Tor Vergata University, Rome, Italy; 10 Volunteers Association of Tor Vergata University Hospital (PTV), Rome, Italy; 11 Clinical Biochemistry and Molecular Biology, Department of Experimental Medicine, Faculty of Medicine, Tor Vergata University, Rome, Italy; 12 Department of Experimental Medicine, Tor Vergata University, Rome, Italy

Correction to: *Scientific Reports* https://doi.org/10.1038/s41598-023-29519-7, published online 13 February 2023

The original version of this Article contained errors in the name of authors Annamaria D'Alessandro, Domenico Ciavardelli, Anna Pastore, Santina Lupisella, Rosa Carmela Cristofaro, Giovina Di Felice, Roberta Salierno, Marco Infante, Alberto De Stefano, Andrea Onetti Muda, Maria Morello, Ottavia Porzio which were incorrectly given as D’Alessandro Annamaria, Ciavardelli Domenico, Pastore Anna, Lupisella Santina, Cristofaro Rosa Carmela, Di Felice Giovina, Roberta Salierno, Infante Marco, De Stefano Alberto, Onetti Muda Andrea, Morello Maria and Porzio Ottavia.

The original Article has been corrected.

