# Supplementary material for: Contribution of vitamin D3 and thiols status to the outcome of COVID-19 disease in Italian pediatric and adult patients
Source: Sci Rep. 2023 Feb 13;13:2504. doi: 10.1038/s41598-023-29519-7 (PMC9925220; doi:10.1038/s41598-023-29519-7)

Supplementary Table 1. Correlation coefficients (ρ) and p values obtained from the Spearman's correlation analysis of clinical features measured in non-survivor adult patients. Significant p-values ($p<0.050$) and the corresponding ρ values are highlighted in yellow.

Spearman ρ	Age	NLR	CRP	D-Dimer	Fibrinogen	IL-6	TNF-α	VD_2	VD_3	Total VD	PTC	Ferritin	LDH	Cys	Cys-Gly	GSH	Hcy
**Age**	1.00																
**NLR**	-0.02	1.00															
**CRP**	-0.19	0.05	1.00														
**D-Dimer**	0.12	0.14	0.14	1.00													
**Fibrinogen**	-0.09	-0.04	0.609	-0.04	1.00												
**IL-6**	-0.21	0.00	0.315	0.19	0.16	1.00											
**TNF-α**	-0.03	-0.04	0.09	0.25	0.10	0.358	1.00										
**VD_2**	-0.06	-0.13	-0.21	-0.09	0.05	-0.18	0.04	1.00									
**VD_3**	-0.07	-0.24	-0.03	-0.12	0.16	-0.06	0.10	0.272	1.00								
**Total VD**	-0.12	-0.17	-0.04	-0.13	0.13	-0.04	0.09	0.333	0.995	1.00							
**PTC**	-0.19	0.22	0.446	0.367	0.382	0.532	0.371	-0.411	-0.300	-0.345	1.00						
**Ferritin**	-0.499	-0.385	0.468	0.08	0.480	0.32	-0.25	0.15	0.32	0.32	0.24	1.00					
**LDH**	-0.02	0.16	0.23	0.356	0.03	-0.01	-0.03	0.20	0.02	0.00	0.14	0.11	1.00				
**Cys**	0.11	-0.07	-0.20	0.05	-0.22	-0.12	-0.02	0.302	0.03	0.07	-0.375	-0.33	-0.02	1.00			
**Cys-Gly**	0.11	0.14	-0.14	-0.12	0.00	0.01	0.310	-0.08	-0.02	-0.02	0.17	-0.433	-0.03	-0.15	1.00		
**GSH**	0.01	-0.26	0.00	-0.12	0.09	-0.01	-0.15	-0.01	-0.05	-0.09	0.13	0.14	-0.04	-0.09	0.24	1.00	
**Hcy**	0.23	0.10	-0.13	-0.05	-0.06	-0.04	0.11	0.05	-0.11	-0.16	0.12	-0.33	-0.05	0.11	0.08	0.11	1.00

Spearman p	Age	NLR	CRP	D-Dimer	Fibrinogen	IL-6	TNF-α	VD_2	VD_3	Total VD	PTC	Ferritin	LDH	Cys	Cys-Gly	GSH	Hcy
**Age**																	
**NLR**	0.89																
**CRP**	0.16	0.69															
**D-Dimer**	0.38	0.31	0.31														
**Fibrinogen**	0.52	0.80	<0.001	0.78													
**IL-6**	0.12	0.98	0.019	0.18	0.25												
**TNF-α**	0.83	0.78	0.49	0.07	0.44	0.010											
**VD_2**	0.68	0.35	0.13	0.52	0.69	0.19	0.78										
**VD_3**	0.62	0.08	0.81	0.41	0.25	0.65	0.46	0.045									
**Total VD**	0.38	0.21	0.75	0.36	0.34	0.77	0.52	0.013	<0.001								
**PTC**	0.20	0.13	0.001	0.011	0.007	<0.001	0.009	0.004	0.041	0.016							
**Ferritin**	0.008	0.05	0.014	0.70	0.011	0.11	0.20	0.45	0.10	0.11	0.25						
**LDH**	0.90	0.28	0.11	0.013	0.82	0.92	0.82	0.18	0.91	0.98	0.36	0.61					
**Cys**	0.40	0.60	0.13	0.73	0.10	0.39	0.89	0.025	0.83	0.61	0.009	0.09	0.92				
**Cys-Gly**	0.43	0.31	0.32	0.39	1.00	0.92	0.020	0.55	0.89	0.87	0.25	0.024	0.82	0.27			
**GSH**	0.93	0.06	0.98	0.37	0.50	0.96	0.27	0.96	0.71	0.51	0.38	0.48	0.76	0.51	0.07		
**Hcy**	0.09	0.45	0.32	0.72	0.67	0.79	0.44	0.71	0.42	0.24	0.40	0.10	0.72	0.44	0.57	0.44	

Supplementary Table 2. Correlation coefficients (ρ) and p values obtained from the Spearman's correlation analysis of clinical features measured in survivor adult patients. Significant p-values ($p<0.050$) and the corresponding ρ values are highlighted in yellow.

Spearman ρ	Age	NLR	CRP	D-Dimer	Fibrinogen	IL-6	TNF-α	VD_2	VD_3	Total VD	PTC	Ferritin	LDH	Cys	Cys-Gly	GSH	Hcy
**Age**	1,00																
**NLR**	0,08	1,00															
**CRP**	0,17	0,382	1,00														
**D-Dimer**	0,21	0,12	0,311	1,00													
**Fibrinogen**	0,20	0,15	0,540	0,13	1,00												
**IL-6**	-0,11	0,426	0,23	0,295	0,21	1,00											
**TNF-α**	0,16	0,14	0,338	0,19	0,18	0,17	1,00										
**VD_2**	-0,06	-0,07	-0,16	-0,23	-0,06	-0,11	0,13	1,00									
**VD_3**	0,08	-0,12	-0,03	-0,06	0,12	0,01	-0,08	0,285	1,00								
**Total VD**	0,09	-0,09	0,05	-0,05	0,18	-0,04	-0,02	0,239	0,929	1,00							
**PTC**	-0,01	0,07	-0,25	0,02	-0,327	0,04	-0,17	0,10	-0,11	-0,16	1,00						
**Ferritin**	0,22	0,488	0,51	0,19	0,385	0,429	0,308	-0,19	-0,25	-0,24	-0,12	1,00					
**LDH**	0,20	0,500	0,51	0,355	0,308	0,446	0,14	0,07	0,02	0,02	-0,15	0,400	1,00				
**Cys**	0,01	-0,08	0,12	0,00	0,06	-0,13	0,16	0,06	-0,03	0,03	-0,02	-0,01	0,21	1,00			
**Cys-Gly**	-0,14	-0,11	-0,06	-0,14	0,01	0,14	0,08	0,09	0,01	0,03	-0,08	-0,07	-0,11	-0,12	1,00		
**GSH**	-0,224	0,12	0,16	-0,18	0,08	0,10	-0,05	-0,06	-0.004	-0.005	-0,06	-0,06	-0,08	-0,07	0,20	1,00	
**Hcy**	-0,09	-0,246	-0,17	-0,01	-0,03	0,03	-0,05	0,15	0,11	0,04	0,09	-0,18	-0,01	0,430	0,20	-0,01	1,00

Spearman p	Age	NLR	CRP	D-Dimer	Fibrinogen	IL-6	TNF-α	VD_2	VD_3	Total VD	PTC	Ferritin	LDH	Cys	Cys-Gly	GSH	Hcy
**Age**																	
**NLR**	0,48																
**CRP**	0,14	0,001															
**D-Dimer**	0,09	0,33	0,012														
**Fibrinogen**	0,07	0,21	0,000	0,29													
**IL-6**	0,36	0,000	0,06	0,020	0,08												
**TNF-α**	0,16	0,23	0,003	0,12	0,12	0,16											
**VD_2**	0,63	0,55	0,18	0,06	0,58	0,36	0,25										
**VD_3**	0,50	0,29	0,77	0,63	0,28	0,93	0,50	0,012									
**Total VD**	0,45	0,46	0,70	0,71	0,11	0,76	0,83	0,036	0,000								
**PTC**	0,97	0,62	0,08	0,89	0,019	0,77	0,23	0,49	0,42	0,25							
**Ferritin**	0,15	0,001	0,000	0,21	0,008	0,005	0,037	0,21	0,09	0,10	0,52						
**LDH**	0,12	0,000	0,000	0,009	0,016	0,001	0,27	0,62	0,88	0,85	0,34	0,011					
**Cys**	0,90	0,48	0,30	0,97	0,60	0,29	0,17	0,58	0,83	0,83	0,88	0,93	0,12				
**Cys-Gly**	0,22	0,35	0,63	0,26	0,91	0,24	0,51	0,46	0,93	0,78	0,57	0,64	0,42	0,30			
**GSH**	0,050	0,31	0,19	0,14	0,51	0,42	0,67	0,62	0.36	0.47	0,67	0,69	0,56	0,52	0,08		
**Hcy**	0,44	0,032	0,14	0,91	0,82	0,79	0,67	0,19	0,35	0,75	0,52	0,24	0,93	0,000	0,09	0,96	

Supplementary Table 3. Correlation coefficients (ρ) and p values obtained from the Spearman's correlation analysis of clinical features measured in children patients. Significant p-values (p<0.050) and the corresponding ρ values are highlighted in yellow.

Spearman ρ	Age	NLR	CRP	D-Dimer	Fibrinogen	IL-6	TNF-α	VD_2	VD_3	Total VD	PTC	Ferritin	LDH	Cys	Cys-Gly	GSH	Hcy
**Age**	1.00																
**NLR**	0.409	1.00															
**CRP**	-0.15	0.27	1.00														
**D-Dimer**	-0.24	-0.184	0.154	1.00													
**Fibrinogen**	0.19	0.40	0.64	0.11	1.00												
**IL-6**	-0.04	0.06	0.05	0.04	-0.08	1.00											
**TNF-α**	0.19	0.06	-0.11	-0.346	0.02	0.03	1.00										
**VD_2**	0.28	-0.06	-0.11	-0.13	-0.15	0.04	0.18	1.00									
**VD_3**	-0.15	0.05	0.05	0.399	0.01	0.21	-0.20	-0.24	1.00								
**Total VD**	-0.16	0.04	0.04	0.399	0.01	0.20	-0.20	-0.23	0.999	1.00							
**PTC**	-0.501	0.05	0.423	0.31	0.22	0.22	-0.18	-0.24	0.491	0.486	1.00						
**Ferritin**	-0.11	0.20	0.454	0.18	0.20	0.10	-0.10	-0.15	0.29	0.29	0.26	1.00					
**LDH**	-0.51	0.00	0.19	0.27	0.00	-0.05	-0.09	-0.17	-0.07	-0.06	0.27	-0.20	1.00				
**Cys**	-0.12	0.02	-0.11	-0.01	-0.07	-0.28	0.01	0.03	-0.01	0.00	0.14	-0.22	0.04	1.00			
**Cys-Gly**	0.16	-0.06	-0.08	-0.28	-0.05	-0.05	-0.06	0.06	-0.19	-0.20	-0.15	-0.01	-0.29	0.465	1.00		
**GSH**	-0.13	0.07	-0.11	-0.10	-0.20	-0.10	0.00	0.00	-0.20	-0.20	-0.08	0.01	0.25	0.04	0.07	1.00	
**Hcy**	0.07	0.09	-0.14	-0.20	-0.15	-0.23	-0.26	0.09	0.03	0.04	0.01	-0.10	0.05	0.594	0.445	0.05	1.00

Spearman p	Age	NLR	CRP	D-Dimer	Fibrinogen	IL-6	TNF-α	VD_2	VD_3	Total VD	PTC	Ferritin	LDH	Cys	Cys-Gly	GSH	Hcy
**Age**																	
**NLR**	0.015																
**CRP**	0.41	0.12															
**D-Dimer**	0.17	0.30	0.38														
**Fibrinogen**	0.28	0.019	<0.001	0.54													
**IL-6**	0.80	0.73	0.79	0.83	0.66												
**TNF-α**	0.28	0.74	0.54	0.045	0.90	0.86											
**VD_2**	0.10	0.73	0.53	0.47	0.38	0.82	0.30										
**VD_3**	0.39	0.78	0.79	0.019	0.95	0.22	0.26	0.16									
**Total VD**	0.37	0.81	0.81	0.019	0.95	0.24	0.26	0.19	<0.001								
**PTC**	0.002	0.80	0.011	0.070	0.20	0.21	0.29	0.17	0.003	0.003							
**Ferritin**	0.55	0.25	0.006	0.32	0.26	0.57	0.57	0.38	0.09	0.10	0.12						
**LDH**	0.002	0.99	0.26	0.12	0.99	0.77	0.61	0.33	0.71	0.72	0.12	0.24					
**Cys**	0.52	0.94	0.55	0.96	0.71	0.14	0.97	0.89	0.97	1.00	0.46	0.25	0.84				
**Cys-Gly**	0.39	0.76	0.68	0.14	0.81	0.78	0.74	0.76	0.32	0.30	0.41	0.94	0.12	0.010			
**GSH**	0.48	0.71	0.58	0.60	0.29	0.61	0.98	0.98	0.29	0.28	0.66	0.94	0.19	0.83	0.72		
**Hcy**	0.71	0.65	0.46	0.30	0.42	0.23	0.16	0.62	0.89	0.85	0.98	0.59	0.81	0.001	0.014	0.78	

Supplementary Figure 1. Unsupervised hierarchical cluster analysis of VD forms and thiols assessed in non-survivors adult patients (NS), survivors adult patients (S), and children patients (C). The figure shows the heatmap in which each column represents a sample and each row a variable. The light red indicates concentration levels above the mean measured concentration. The light blue color represents concentrations lower than the mean measured concentration. Data were log transformed,mean centered, and scaled. Euclidian distances were used with the Ward's clustering algorithm.

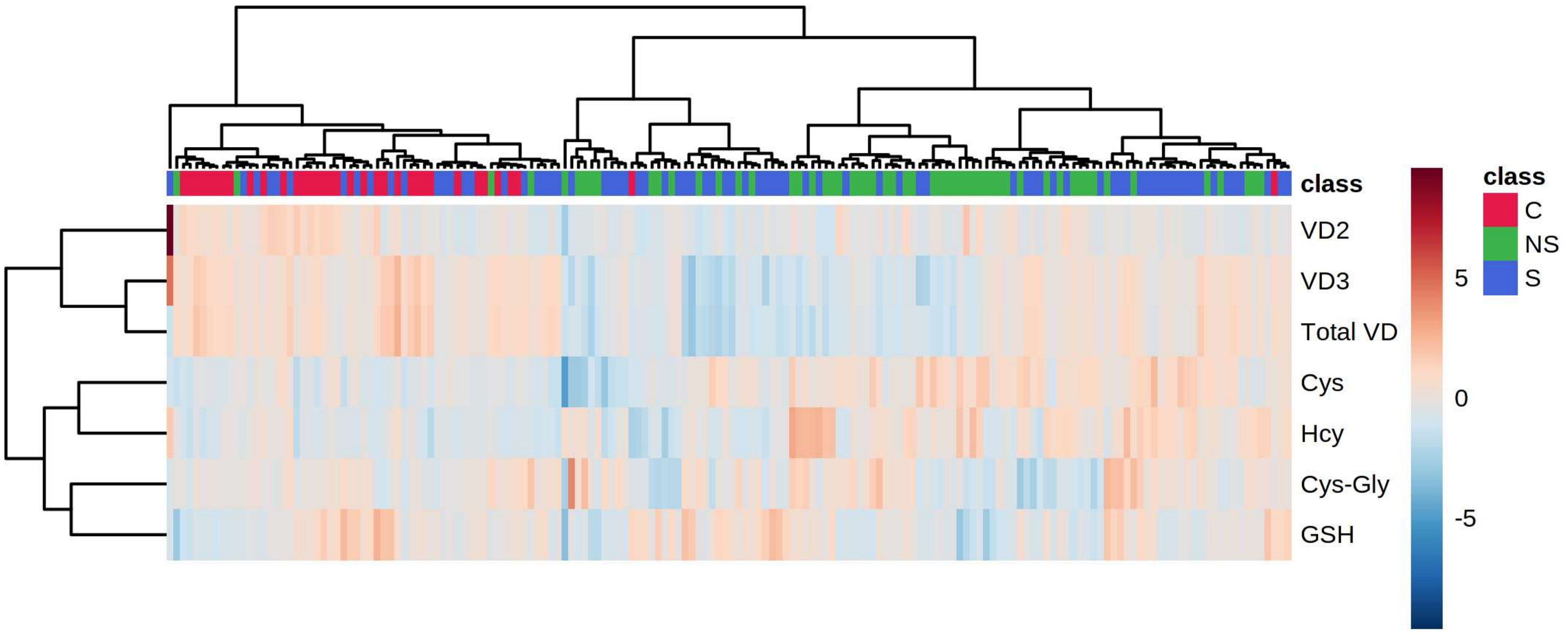

Supplement: Supplementary file 1 — Supplementary Information. [file 41598_2023_29519_MOESM1_ESM.pdf]
